# Dihydrotanshinone I protects human chondrocytes and alleviates damage from spontaneous osteoarthritis in a guinea pig model

**DOI:** 10.1038/s41598-023-48902-y

**Published:** 2023-12-04

**Authors:** Yan-Zhuo Zhang, Zhen-Jie Wei, Shu-Nan Yu, Xin-Yu Wang, Ying Wang, Cheng-Ai Wu, Xu Jiang

**Affiliations:** 1 National Center for Orthopaedics, Department of Molecular Orthopaedics, Beijing Research Institute of Traumatology and Orthopaedics, Beijing Jishuitan Hospital, Capital Medical University, Beijing, 100035 People’s Republic of China; 2 National Center for OrthopaedicsDepartment of Orthopaedics, Beijing Research Institute of Traumatology and Orthopaedics, Beijing Jishuitan Hospital, Capital Medical University, Beijing, 100035 People’s Republic of China

**Keywords:** Drug discovery, Diseases

## Abstract

Osteoarthritis (OA) is the most common degenerative joint disease. Currently, no satisfactory pharmacological treatment exists for OA. The potential anti-inflammatory properties of Dihydrotanshinone I (DHT) have been reported, but its effects on OA are unclear. In this study, we assess the impact of DHT on the viability of human chondrocytes in vitro. We then use a guinea pig model to investigate the effects of DHT on knee osteoarthritis progression. Twelve-week-old Dunkin Hartley guinea pigs spontaneously developing OA were intraperitoneally injected with different doses of DHT for eight weeks. Micro-CT analysis was performed on the subchondral bone in the knee, and histological assessment of the knee joint was done using stained sections, the ratio of hyaline to calcified cartilage, and Mankin scores. DHT successfully restored IL-1β-induced decreases in cell viability in human primary chondrocytes. In the guinea pig model, intraperitoneal injections of DHT ameliorated age-induced OA, effectively reduced the expression level of two cartilage metabolism-related genes (*ADAMTS4* and *MMP13*) and decreased the inflammatory biomarker IL-6 in the serum of guinea pigs developing spontaneous osteoarthritis. These findings demonstrate DHT’s protective effects on chondrocytes and suggest that it alleviates cartilage degradation and proteoglycan loss in OA.

## Introduction

Osteoarthritis (OA) is a chronic degenerative bone and joint disease that is more prevalent among the middle-aged and elderly. Patients suffer significant daily life challenges due to progressive joint pain, swelling, joint deformity, and stiffness^1–3^. Despite extensive research, the pathogenesis of OA remains enigmatic, and there is an urgent need to identify the key molecular mechanisms underlying the initiation and progression of OA. Depending on the stage of the disease, current treatment strategies focus on chronic pain relief and joint mobility preservation by viscosupplementation injection or physiotherapy for early-stage OA^2,4–6^, and total joint replacement surgery for end-stage OA^6–8^. However, there is still a lack of disease-modifying OA drugs or agents to improve joint homeostasis^6^. A substantial amount of time and effort is required to investigate potential therapeutic drugs that may alter the degenerated joint phenotype in OA.

Articular cartilage is a highly organized avascular fibrous connective tissue that serves as a shock absorber with low-friction and low-wear properties, ensuring smooth joint articulation^9,10^. It is composed of a complex extracellular matrix (ECM) primarily comprised of type II collagen and aggrecan, a large aggregating chondroitin-sulfate proteoglycan, as well as sparsely distributed specialized cells called chondrocytes^11^. As the only cells found in cartilage, chondrocytes play a significant role in the development and maintenance of the ECM, which is in a dynamic state of equilibrium between synthesis and degradation^12,13^. Matrix metalloproteinase 13 (MMP13) and a disintegrin and metalloproteinase with thrombospondin motif 4 (ADAMTS4) are considered the most critical ECM catabolic enzymes associated with the degradation of collagen and aggrecan, respectively, in OA^14,15^. Dysregulation of these ECM-degrading enzymes can disrupt the balance between ECM synthesis and degradation, leading to progressive pathological destruction in cartilage^16^.

Current models of knee osteoarthritis (KOA) involve surgical methods, such as anterior cruciate ligament disconnection and knee meniscus tear^17,18^. However, these models fail to replicate the progressive degeneration naturally occurring in a KOA^19^. To address this issue, we used an animal model in our study. The Dunkin Hartley guinea pig model of KOA replicates the natural progression of KOA, allowing for mechanistic studies of the cartilage degeneration typically observed in human KOA^1,19,20^. Moreover, lesions in Dunkin Hartley guinea pigs spontaneously developing KOA correspond to their age and weight^1,21,22^.

The traditional Chinese medicine (TCM) herb *Salvia miltiorrhiza* Bunge (commonly known as Danshen) is well-established in the treatment of hepatitis, amenorrhea, bleeding, and cardiovascular problems^23^. The main active ingredients in *S. miltiorrhiza* include hydrophilic and lipophilic fractions^24^. The hydrophilic fractions include key ingredients such as salianolic acid derivatives and diterpenoids, while the lipophilic fractions contain diverse tanshinones^25^. Among these tanshinones, dihydrotanshinone I (DHT) has received considerable attention due to its wide range of biological effects^26^. DHT is an abietane diterpenoid compound with the molecular formula: C_18_H_14_O_3_. It is a natural compound extracted from *S. miltiorrhiza*, also known as the Chinese sage, red sage root, or the Chinese herb Dan Shen. In addition to its ability to reverse multidrug resistance and promote vasorelaxation of coronary arteries in rats, DHT has shown promise as a tumor suppressor by inducing cytotoxicity and reducing angiogenesis. Furthermore, DHT has demonstrated anti-inflammatory properties both in vitro and in vivo^26–31^. Potential anti-inflammatory properties of DHT have been reported for acute kidney injury^27^, adipose tissue inflammation^30^ and NLRP3 inflammasome^31^. Yuan et al.^27^ demonstrated that DHT exerted anti-inflammatory effects by inhibiting the secretions of inflammatory cytokines and the activation of the NF-κB pathway in vitro and by alleviating LPS-induced acute kidney injury in vitro. Similarly, Wang et al.^28^ revealed that DHT suppressed the expression of the *NF-κB* reporter gene and TNF-α-induced phosphorylation of IκBα and p65. They also found that DHT inhibited the expressions of NF-κB target genes including TNF-α, IL-6, and MCP1^28^. However, research is limited on whether DHT can prevent osteoarthritis and its underlying mechanism. Therefore, this study aimed to investigate the potential protective effects of DHT on IL-1β-induced chondrocyte inflammation and its therapeutic effect in Dunkin Hartley guinea pigs during the development of OA.

## Materials and methods

### Human cell study

#### Cell culture and treatment

Human primary chondrocytes (Bena Culture Collection) were cultured in DMEM (Thermo Fisher Scientific) supplemented with 20% heat-inactivated FBS and 1% antibiotics (Gibco; Thermo Fisher Scientific) and maintained at 37 °C in a humidified atmosphere with 5% CO_2_. Cells were then seeded into a 96-well plate at a density of 3000 cells/well and incubated overnight. DHT (Chengdu Biopurify Phytochemicals) was dissolved in DMSO under a laminar flow hood and sterilized using a filter. After 3 h of incubation, different concentrations (0, 0.1, 1, 2, 4, 8 μM) of DHT were added to wells, followed by the addition of IL-1β (10 ng/ml; Sigma) or a blank control. All in vitro experiments were repeated at least three times.

#### Cell viability assay

Briefly, cells were seeded into 96-well plates at a density of 3 × 10^3^ cells/well and incubated overnight. Following the treatment, 10 µl of Cell Counting Kit-8 (CCK-8; Invitrogen) was added to each well, and cells were further incubated for 2 h at 37 °C. The absorbance of each well at 450 nm was measured using a microplate reader. The cell viability of the control group was set to 100%.

### Guinea pig study

#### Animals

Female Dunkin Hartley (DH) guinea pigs (n = 56) aged 4 weeks were housed in a specific pathogen-free animal room maintained at a temperature of 18–22 °C, a humidity range of 40–70%, and a 12-h light/dark cycle. All animal experiment procedures were approved by the Beijing Jishuitan Hospital Animal Care and Use Committee, Beijing, China, and all methods followed the institutional guidelines for the care and use of animals. The study is reported following the ARRIVE guidelines (https://arriveguidelines.org). We measured the body weight (kg) of DH guinea pigs in each month-age group and drew the weight-month-age curve for each group.

#### Study design

Guinea pigs were given ad libitum access to sterilized chow and water. Eight of the 4-week-old guinea pigs acquired were sacrificed and sampled for microcomputed tomography (micro-CT) imaging and histological evaluation. After 8 weeks of acclimatization, a further eight guinea pigs aged 12 weeks were sacrificed for micro-CT and histology analyses. The remaining 40 were divided into five groups (n = 8 animals/group): (1) Normal group, no treatment; (2) Vehicle group, receiving a vehicle control of DMSO; (3) Low dose group, receiving intraperitoneal injection of DHT (5 mg/kg); (4) Medium dose group, receiving intraperitoneal injection of DHT (10 mg/kg); and (5) High dose group, receiving intraperitoneal injection of DHT (25 mg/kg). Animals received weekly DHT or vehicle control injections intraperitoneally for 4 or 8 weeks. All animals were sacrificed after the final injection at 21 weeks of age.

#### Histology and micro-CT evaluation

To collect tissue samples, guinea pigs were first anesthetized by an intramuscular injection of 0.25 mg/kg dexmedetomidine and 25 mg/kg ketamine, and then blood samples collected from the heart. The plasma was allowed to stand at room temperature for 4 h, and the supernatant serum was separated by centrifugation.

Animals were then sacrificed, and the left knee joint from each guinea pig was subjected to micro-CT scanning. Before scanning, the knee joints were kept in the guinea pigs' resting position to ensure consistent imaging. The proximal tibiae were scanned using a high-resolution micro-CT system (SkyScan 1176, Aartselaar, Belgium) to quantify the subchondral bone plate thickness (defined as starting from the calcified cartilage-bone junction and ending at the marrow space) and the micro-architecture of the subchondral trabecular bone^1,32^. A series of 21 μm tomographic scans were performed at 70 kV. A 3D reconstruction was done using the system software and the following parameters were analyzed: bone volume ratio (BV/TV), the thickness of the subchondral bone plate (SBP), and the structure model index (SMI).

After the micro-CT scan, the left knee joint was isolated, fixed in 4% paraformaldehyde, washed with water, decalcified with 30% formic acid, dehydrated with ethanol, and embedded in paraffin. Five-micrometer sections were acquired at 50-µm intervals to obtain a series of coronal sections of the entire joint. The sections were stained as follows: hematoxylin–eosin (HE; Solarbio), safranin O-fast green (Safranin O), and Alcian. The degree of OA was evaluated by two investigators blind to treatment using the ratio of hyaline (HC)/calcified cartilage (CC) and Mankin scores for analysis^33^.

#### qRT-PCR

To assess *MMP13* and *ADAMTS4* expression levels, total RNA from the cartilage samples was extracted with the RNA isolator Total RNA Extraction Reagent Isolator (TRIzol method, Beyotime, China). cDNA was reverse transcribed using the Hiscript II Q RT Supermix from 1 μg of total RNA. Then, qPCR was performed and the relative expression analyzed by the △△Ct method and normalized to the expression level of *GAPDH*. The primer sequences used for PCR are: GAPDH (F): 5′-TCTCCTCTGACTTCAACAGCGAC-3′, (R): 3′-CCCTGTTGCTGTAGCCAAATTC-5′; MMP-13 (F):5′-CTTCCCAACCGTATTGATGCT-3′, (R): 3′-CTGGTTTCCTGAGAACAGGAG-5′; ADAMTS4 (F): 5′-TGACAAGTGCATGGTGTGTG-3′, (R): 3′-AGTAAATGTTCCGAGGGCCA-5′.

#### Enzyme-linked immunosorbent assay (ELISA)

Levels of IL-6 in guinea pig serum were detected using the ELISA kit provided by Nanjing Xinfan Biology. Absorbance at 450 nm was measured using a microplate reader from Molecular Devices and concentrations were calculated using a standard curve.

### Statistical analysis

All experiments were performed with at least three independent biological replicates. Data are presented as the mean ± SD. Statistical analyses were performed using a one-way or two-way ANOVA with Tukey’s post-hoc test for multiple comparisons. *P* < 0.05 was considered statistically significant.

### Ethics approval and consent to participate

All animal experiment procedures were approved by the Beijing Jishuitan Hospital Animal Care and Use Committee, Beijing, China, and the study is reported in accordance with ARRIVE guidelines (https://arriveguidelines.org).

## Results

### Human cell study

#### DHT restored IL-1β-induced decrease in cell viability in human primary chondrocytes

**Figure 1:**
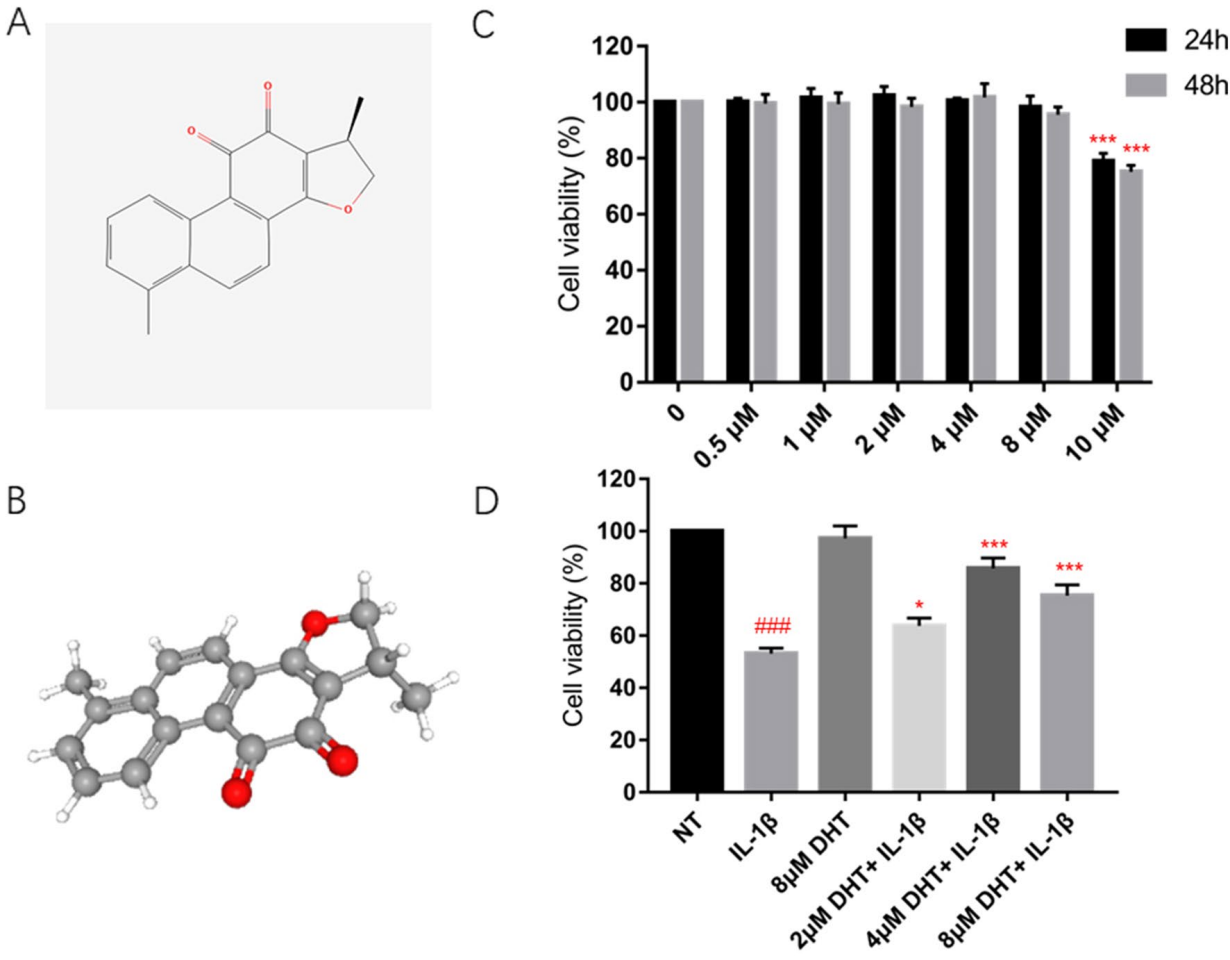
DHT alleviated the IL‐1β-induced decrease in cell viability of the human primary chondrocytes. (**A**) The 2D chemical structure of DHT. (**B**) The 3D chemical structure of DHT. (**C**) Cell viability of the human primary chondrocytes 24 h and 48 h after treatment with DHT in concentrations of 0, 0.5, 1, 2, 4, 8, and 10 μM. (**D**) Cell viability of the human primary chondrocytes after treatment with DHT of 2, 4, and 8 mM, followed by 3 h incubation with IL-1β (10 ng/ml). The data are presented as the mean ± SEM. ^#^*P* < 0.05, ^##^*P* < 0.01, and ^###^*P* < 0.001 versus control; *P < 0.05, **P < 0.01, and ***P < 0.001 versus vehicle group.

DHT appears as a red, odorless powder and is soluble in both water and ethanol. Its two-dimensional and three-dimensional structures are illustrated in Fig. 1A,B, respectively. We first investigated the impact of DHT on human primary chondrocytes under resting conditions. Increasing concentrations of DHT (ranging from 0 to 8 mM) had no cytotoxic effects on resting human primary chondrocytes at 24 or 48 h after treatment (Fig. 1C), indicating the safety of applying DHT to human primary chondrocytes. Subsequently, we examined the impact of DHT on IL-1β-induced decrease in chondrocyte activity (Fig. 1D). DHT at concentrations of 2, 4, and 8 μM restored chondrocyte activity to varying degrees.

### Guinea pig study

#### Natural progression of osteoarthritis in the guinea pig model at different ages

**Figure 2:**
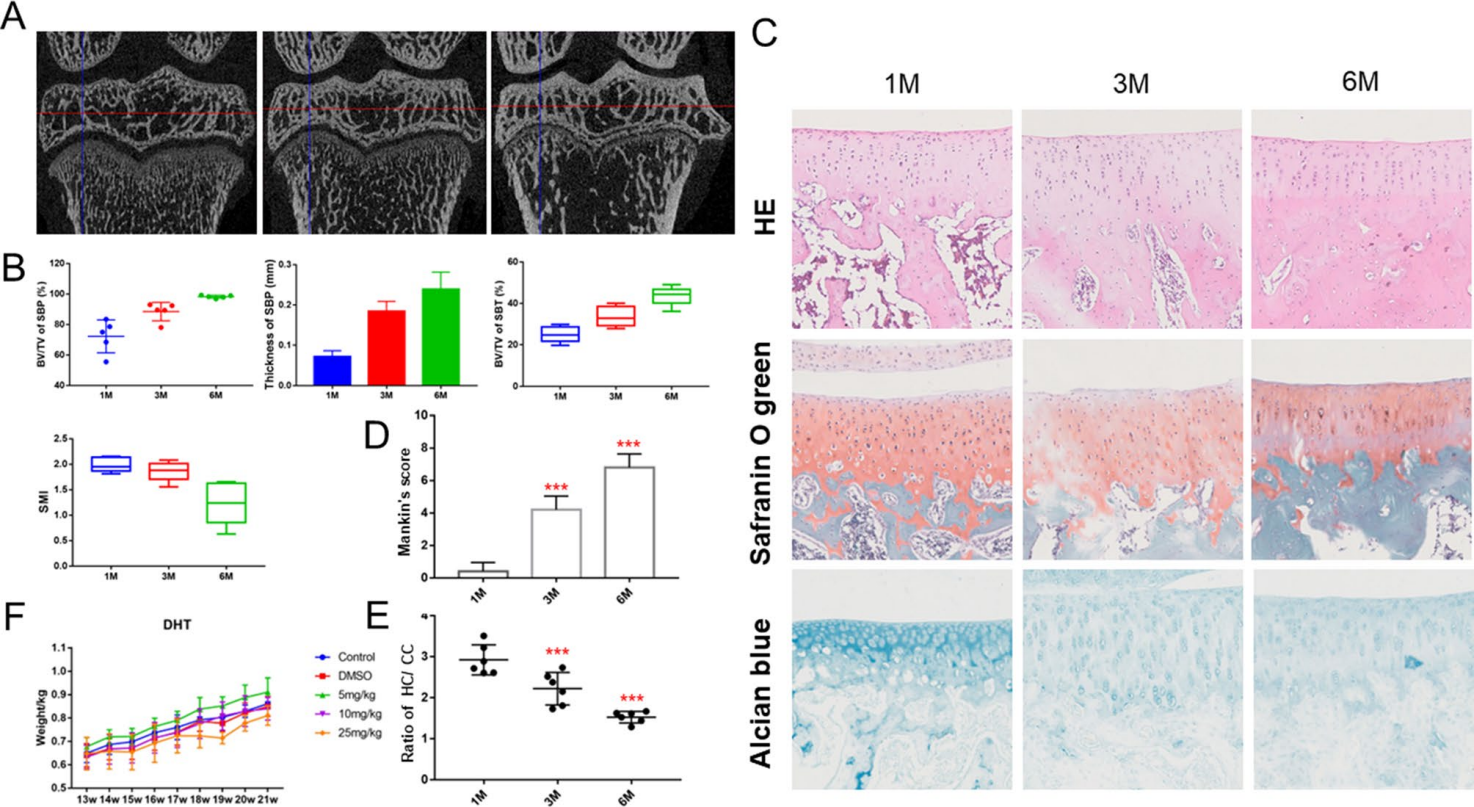
Stages of spontaneous knee osteoarthritis in a guinea pig model. (**A**) Representative sagittal images of the medial subchondral bone in the left tibial plateau. (**B**) Micro-CT parameters of the medial subchondral bone in the left tibial plateau of the guinea pig model. (**C**) Representative images of the subchondral bone at the medial tibial plateau after staining with hematoxylin–eosin, Safranin O, and Alcian. Original magnification × 20. (**D**, **E**) The ratio of Mankin score and HC/CC. (**F**) Total body weight curve for each age group of guinea pigs. The data are presented as the mean ± SEM. *P < 0.05, **P < 0.01, and ***P < 0.001 versus 1-month group.

Micro-CT was used to obtain images and measures of the subchondral bone at the medial tibial plateau in guinea pigs (Fig. 2A). As age increased, we observed a notable thickening in the subchondral bone plate (SBP) and an expansion of the trabecular bone width (Fig. 2B). The zone of bone density reduction in the proximal tibia gradually increased with age and became surrounded by trabecular bone. As guinea pigs aged, the subchondral trabecular bone (SBT) in the weight-bearing area collapsed and fused into pieces or clumps that merged with the adjacent subchondral bone plate (Fig. 2B). This phenomenon was observed in the weight-bearing area of the proximal tibia at 6 months. The subchondral trabecular collapse and fusion observed in our model are very similar to the subchondral bone changes observed in human KOA. Histological staining was conducted on the medial compartment of articular cartilage in guinea pigs at ages 1, 3, and 6 months (Fig. 2C). Guinea pigs at 1 and 3 months of age had a relatively normal tibial surface with chondrocytes evenly distributed throughout the matrix. In contrast, 6-month-old guinea pigs had a significant loss of proteoglycans, extending deep into the tibial cartilage with regions of chondrocyte hypocellularity within the extracellular matrix (ECM). Histologic grading revealed that 6-month-old guinea pigs also had a significantly lower ratio of HC/CC and increased Mankin scores compared to the 1-month-old group (Fig. 2D,E). The body weight of Dunkin–Hartley guinea pigs also increased with age, but there was no significant difference in weight between the same age groups (Fig. 2F).

#### DHT alleviated age-related deterioration of the subchondral bone in tibial plateau

**Figure 3:**
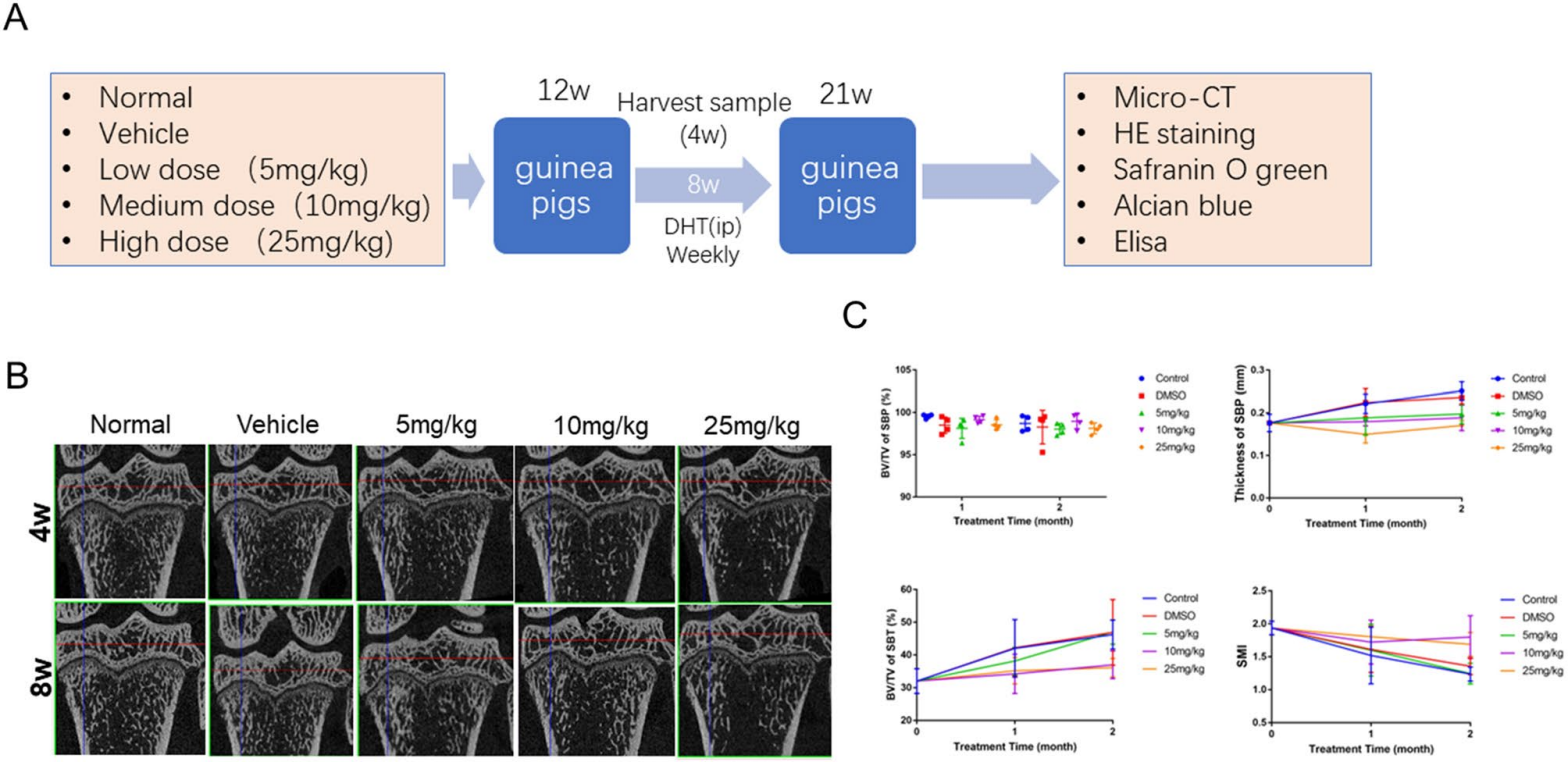
DHT alleviated age-related deterioration of the subchondral bone in the tibial plateau of the guinea pig model. (**A**) Study design. (**B**) Representative sagittal images of the medial subchondral bone in the left tibial plateau. (**C**) Micro-CT parameters of the medial subchondral bone in the left tibial plateau of the guinea pig model. The data are presented as the mean ± SEM. ^#^*P* < 0.05, ^##^*P* < 0.01, and ^###^*P* < 0.001 versus control; *P < 0.05, **P < 0.01, and ***P < 0.001 versus vehicle group.

Having validated the protective effects of DHT on chondrocyte viability in human cells in vitro, we used the guinea pig model to investigate the effect of DHT on articular cartilage and subchondral bone in vivo. The study design of the animal experiments is detailed in Fig. 3A. Using micro-CT imaging, we identified significant changes in the subchondral bone of the medial tibial plateau in both control and vehicle groups with the progression of OA. These changes were attenuated by intraperitoneal injections of DHT at medium and high doses (10 and 25 mg/kg) (Fig. 3B). Additionally, a quantitative analysis of the micro-CT data showed that DHT exhibited an antagonistic effect on measures such as BV/TV of SBT, thickness of SBP, and SMI (Fig. 3C). Thus, treatments with DHT effectively mitigated age-related degradation of the subchondral bone in the tibial plateau of the guinea pig model.

#### DHT mitigated age-related destruction of the articular cartilage in the medial tibial plateau

**Figure 4:**
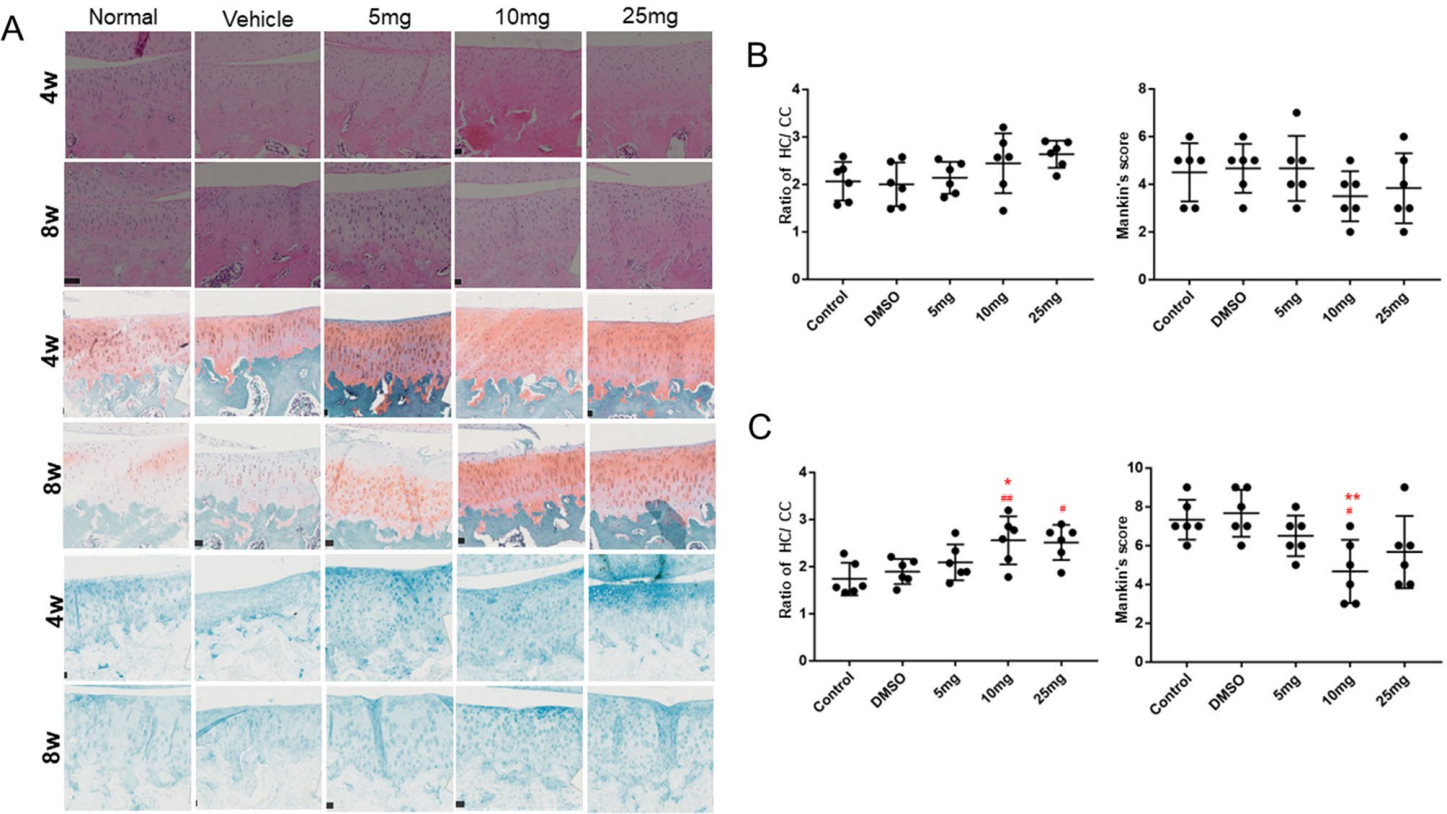
DHT alleviated age-related deterioration of the articular cartilage in the tibial plateau of the guinea pig model. (**A**) Representative images of hematoxylin–eosin, Safranin O, and Alcian staining. Original magnification × 20. (**B**) The ratio of HC/CC and Mankin score for the 4-week treatment group. (**C**) The ratio of HC/CC and Mankin score of the 8-week treatment group. Data are presented as the mean ± SEM. ^#^*P* < 0.05, ^##^*P* < 0.01, and ^###^*P* < 0.001 versus control; *P < 0.05, **P < 0.01, and ***P < 0.001 versus vehicle group.

We next performed HE, Safranin O, and Alcian staining to assess the impact of DHT on the articular cartilage of the medial tibial plateau in the guinea pig model (Fig. 4A). As shown in Fig. 4B, the control and vehicle groups both exhibited age-related defects on the articular cartilage surface and varying degrees of proteoglycan loss. A 4-week treatment of DHT did not lead to a significant improvement in articular cartilage damage and proteoglycan loss relative to the control and vehicle groups. However, an 8-week treatment of DHT, particularly at medium and high doses (10 or 25 mg/kg) significantly reduced both deterioration in the articular cartilage and proteoglycan loss (Fig. 4C). The ratio of HC/CC and Mankin scores were used to further assess the articular cartilage status and confirmed that DHT alleviated age-related destruction of the articular cartilage on the tibial plateau and prevented proteoglycan loss in the guinea pig model (Fig. 4C).

#### DHT reduced expression levels of osteoarthritis markers and inflammatory biomarkers

**Figure 5:**
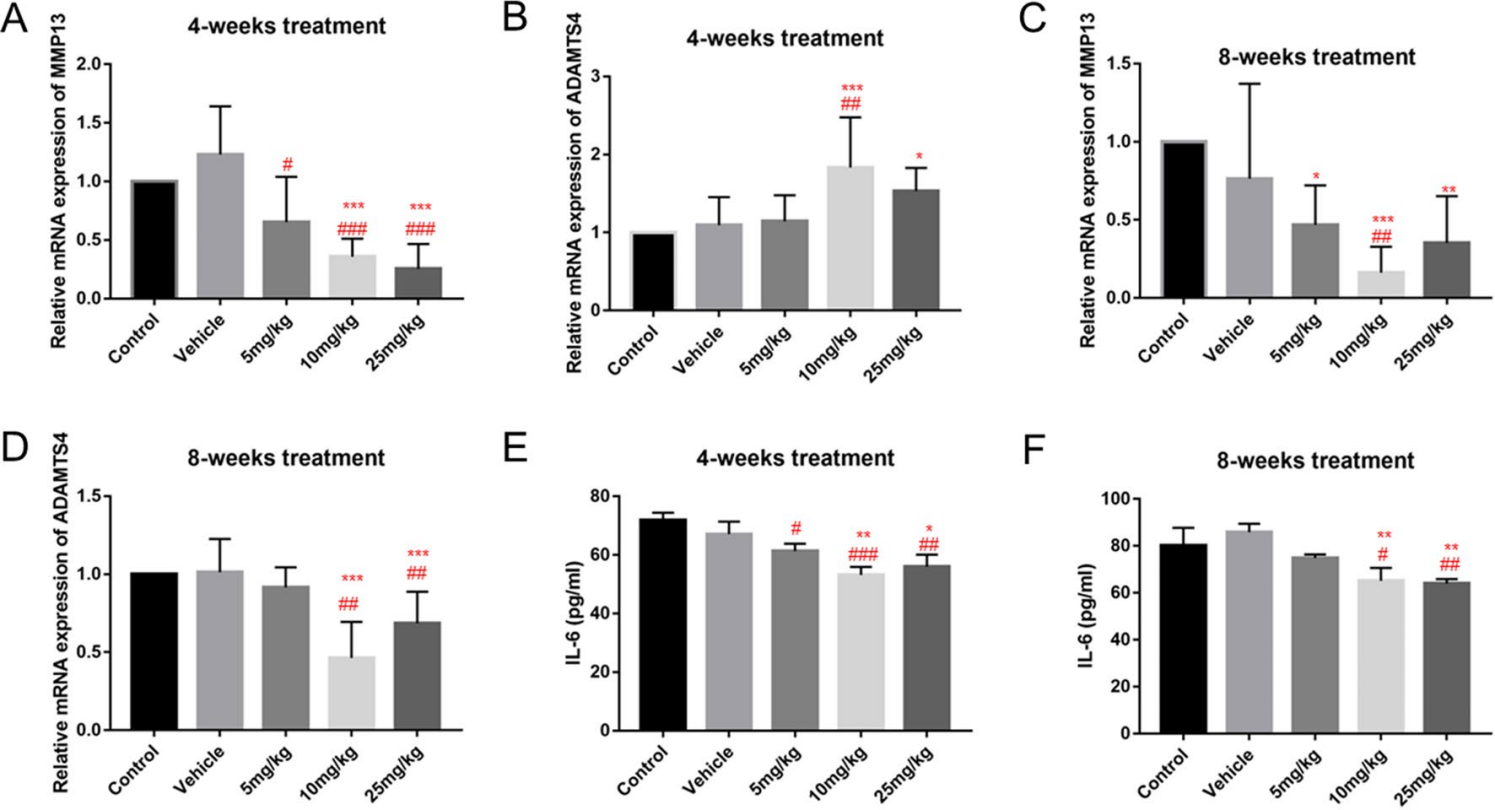
DHT reduced the expression levels of osteoarthritis and inflammatory markers. (**A**–**D**) The mRNA expression levels of *MMP13* and *ADAMTS4* measured using qRT-PCR. (**E**, **F**) The expression levels of IL-6 in serum detected by ELISA. Data are presented as the mean ± SEM. ^#^*P* < 0.05, ^##^*P* < 0.01, and ^###^*P* < 0.001 versus control; *P < 0.05, **P < 0.01 and ***P < 0.001 versus vehicle group.

We used qRT-PCR to measure expression levels of catabolism-related genes *MMP13* and *ADAMTS4,* which play crucial roles in cartilage metabolism within the articular cartilage. Compared to the control and vehicle groups, samples from guinea pigs treated with DHT for 8 weeks, specifically in medium and high doses (10 or 25 mg/kg), exhibited reduced expression levels of *MMP13* and *ADAMTS4* (Fig. 5C,D). However, in the 4-week treatment group, the expression level of *MMP13* was decreased while *ADAMTS4* was increased (Fig. 5A,B). Additionally, we performed ELISA on serum samples obtained from whole blood. Compared to the control and vehicle groups, samples from guinea pigs that received intraperitoneal injections of DHT for 4 and 8 weeks, particularly at medium and high doses (10 or 25 mg/kg), exhibited a reduction in the expression of the inflammatory marker IL-6 (Fig. 5E,F). These findings collectively suggest that DHT treatment effectively suppressed catabolism in articular cartilage and reduced the levels of the inflammatory marker IL-6.

## Discussion

OA is characterized by progressive destruction of articular cartilage, primarily due to the loss of chondrocytes^3^. Chondrocytes are the only cells found in cartilage and play a vital role in maintaining a homeostatic equilibrium between the production and degradation of cartilage ECM^6^. Under the stimulation of inflammatory cytokines, the remaining chondrocytes exhibit an abnormal catabolic phenotype that leads to an imbalance between degradation and synthesis of ECM^8^. Besides the well-characterized matrix metalloproteinases (MMPs), ADAMTSs are the principal proteases expressed in cartilage^9^. Specifically, ADAMTS4, which shows marked changes in expression during OA, is emerging as a promising potential therapeutic target for OA treatment^9,15^.

Guinea pigs are commonly used as a model for investigating naturally occurring age-related OA. Several in vivo models using guinea pigs have been utilized to replicate the initiation of OA and monitor its progression^1,19^. The model used in the present study exhibits specific OA symptoms, such as cartilage deterioration, the growth of marginal osteophytes, and alterations to the subchondral bone. Age-related increases in disease severity start at 3 months with mild OA and ultimately advance to a state of moderate to severe OA at 18 months^1,34^. In this study, micro-CT and immunohistochemical analyses were conducted on guinea pigs aged 1 month, 3 months, and 6 months. In addition, we monitored weight changes in these guinea pigs throughout the study period. It has been documented in previous studies that weight can have a direct impact on the onset and progression of OA due to increased load on the joints^17,18^. Our research results indicate that the severity of OA is influenced by age, and weight significantly increases with age, which may be one of the factors inducing OA.

*Salvia miltiorrhiza*, commonly known as Danshen, is widely used for its anti-inflammatory properties^35^. Among the various components extracted from *S. miltiorrhiza*, DHT is a fat-soluble compound that exhibits anti-inflammatory effects. It is well established that DHT shows strong anti-oxidative and anti-inflammatory properties in inflammation and various other diseases^28,36^. In the current study, in vitro experiments demonstrated that DHT mitigates IL-1β-induced damage to human primary chondrocytes. However, further investigation is needed to fully elucidate the specific underlying mechanism.

In our guinea pig model, we took measures to eliminate the potential influence of the solvent itself on OA by including both control and vehicle groups. We further used micro-CT imaging and analysis to examine the subchondral bone medial to the tibial plateau. Significant damage to the subchondral bone in the control and vehicle groups was observed. This damage was ameliorated in groups receiving intraperitoneal injections of DHT at medium (10 mg/kg) and high (25 mg/kg) doses. It has been previously established that degeneration of the articular cartilage plays a key role in the development of OA. Histological staining revealed age-related partial defects on the articular cartilage surface and varying levels of proteoglycan loss in the control and vehicle groups. Intraperitoneal injections of DHT at medium (10 mg/kg) and high (25 mg/kg) doses significantly reduced the degradation of the articular cartilage and proteoglycan loss relative to the control and vehicle groups. Another characteristic of OA is increased catabolism and decreased anabolism in chondrocytes. Our results further provide evidence that DHT protects cartilage by regulating the activity of important proteolytic enzymes involved in KOA, such as ADAMTS4. Previous studies have demonstrated the significant role of ADAMTS4 and ADAMTS5 in OA development^37^. In addition, MMPs contribute to this process by degrading collagen^38^. The decrease in the secretion of these enzymes by cartilage after treatment with DHT suggests that DHT acts anti-proteolytically by regulating the expression of MMP13. We also assessed the expression of the inflammatory marker IL-6^38^. Compared to the control and vehicle groups, samples from guinea pigs given intraperitoneal injections of DHT at medium and high doses (10 or 25 mg/kg) exhibited decreased expression of IL-6.

There were some limitations to this study. The study focused on the protective effects of DHT on knee osteoarthritis, without conducting a comprehensive exploration of the underlying mechanisms, including the effect of DHT on inflammatory signaling. Future investigations should aim to elucidate how DHT influences extracellular matrix molecules, metalloproteinases, and inflammatory cytokines, as these factors have been suggested to play key roles in the progression of OA.

## Conclusions

In conclusion, the findings of this study suggest that DHT can restore IL-1β-induced decrease in chondrocyte activity, highlighting its protective effects on chondrocytes. This study is the first to demonstrate the protective effects of DHT in preserving articular cartilage integrity and preventing proteoglycan loss in a guinea pig model of age-related cartilage deterioration in the tibial plateau. These results underscore the potential of DHT as a promising therapeutic option for the prevention and treatment of OA.

## Data Availability

All data in the current study are available from Cheng-Ai Wu author upon reasonable request.

## Abbreviations

ADAMTS4: A disintegrin and metalloproteinase with thrombospondin motifs 4
BV/TV: Bone volume fraction
DHT: Dihydrotanshinone I
ECM: Extracellular matrix
HC/CC: Hyaline/calcified cartilage
IL-6: Interleukin-6
Micro-CT: Micro-computed tomography
MMP13: Matrix metallopeptidase-13
OA: Osteoarthritis
OARSI: Osteoarthritis Research Society International
SMI: Structure model index
